# *emle-engine*: A Flexible Electrostatic Machine Learning Embedding Package for Multiscale Molecular Dynamics Simulations

**DOI:** 10.1021/acs.jctc.4c00248

**Published:** 2024-05-28

**Authors:** Kirill Zinovjev, Lester Hedges, Rubén Montagud Andreu, Christopher Woods, Iñaki Tuñón, Marc W. van der Kamp

**Affiliations:** † Departamento de Química Física, Universidad de Valencia, 46100 Burjassot, Spain; ‡ School of Biochemistry, University of Bristol, Biomedical Sciences Building, University Walk, Bristol BS8 1TD, U.K.; § Research Software Engineering, Advanced Computing Research Centre, 31 Great George Street, Bristol BS1 5QD, U.K.

## Abstract

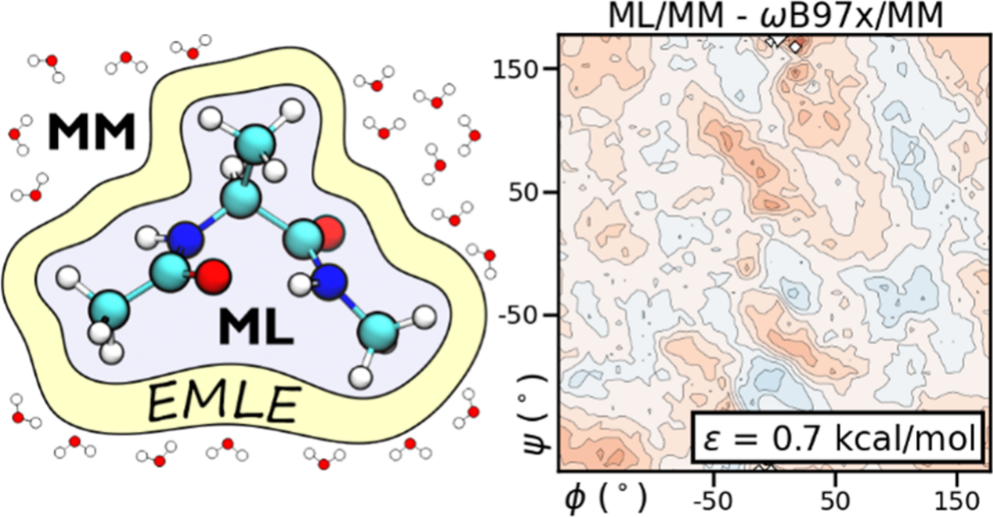

We present in this work the *emle-engine* package (https://github.com/chemle/emle-engine)—the implementation of a new machine learning embedding scheme for hybrid machine learning potential/molecular-mechanics (ML/MM) dynamics simulations. The package is based on an embedding scheme that uses a physics-based model of the electronic density and induction with a handful of tunable parameters derived from *in vacuo* properties of the subsystem to be embedded. This scheme is completely independent of the *in vacuo* potential and requires only the positions of the atoms of the machine learning subsystem and the positions and partial charges of the molecular mechanics environment. These characteristics allow *emle-engine* to be employed in existing QM/MM software. We demonstrate that the implemented electrostatic machine learning embedding scheme (named EMLE) is stable in enhanced sampling molecular dynamics simulations. Through the calculation of free energy surfaces of alanine dipeptide in water with two different ML options for the *in vacuo* potential and three embedding models, we test the performance of EMLE. When compared to the reference DFT/MM surface, the EMLE embedding is clearly superior to the MM one based on fixed partial charges. The configurational dependence of the electronic density and the inclusion of the induction energy introduced by the EMLE model leads to a systematic reduction in the average error of the free energy surface when compared to MM embedding. By enabling the usage of EMLE embedding in practical ML/MM simulations, *emle-engine* will make it possible to accurately model systems and processes that feature significant variations in the charge distribution of the ML subsystem and/or the interacting environment.

## 1 Introduction

Hybrid quantum-mechanics/molecular-mechanics (QM/MM) potentials are the state-of-the-art approach for simulating chemical transformations in condensed phases.^1−5^ By combining a wave function or density function theory (DFT)-based Hamiltonian for a small portion of the system (the QM region), while describing the rest with a computationally efficient molecular mechanics force field, one can consider simulation systems consisting of >10^5^ atoms while still being able to describe electronic rearrangements and bond formation/breaking in the region of interest. However, the performance bottleneck of QM/MM simulations still lies in the QM method of choice. Thus, even for relatively small QM regions (≈10^2^ atoms) and using a relatively efficient (nonhybrid) DFT functional, the time scale of QM/MM molecular dynamics (MD) simulations is typically limited to nanoseconds at best.

A solution to the above-mentioned limitation could come from recent advances in the development of machine-learned molecular potentials (MLPs).^6−9^ The promise of MLPs is to provide molecular energies at near-QM precision and at near-MM cost. Therefore, by replacing an expensive QM Hamiltonian with a cheap ML alternative, one could reach the microseconds scale with the resulting “ML/MM” MD simulations. Unfortunately, even if an ML potential for the QM region of interest is readily available, it is generally not possible to use it with existing QM/MM engines in a “plug-and-play” manner as one would do with an arbitrary QM Hamiltonian. In state-of-the-art QM/MM, the interaction between QM and MM regions is treated with electrostatic embedding: the polarization of the QM part by the MM environment is treated explicitly, by obtaining the electronic density of the QM part in the presence of MM point charges. In contrast to QM methods, ML potentials are generally only trained to predict the total energy of the system *in vacuo*, without considering any external electric fields and without providing a description of the electronic density. Therefore, to employ a pretrained ML potential in an existing QM/MM implementation, one would have to resort to a much simpler “MM embedding” where the electrostatic interaction between the QM and MM region is based on placing fixed charges on the QM atoms, making it appear as “just another MM region” to the rest of the system. This approach is currently used, for example, to enable ML/MM simulations in OpenMM.^10,11^ Alternatively, one can explicitly train the ML model to predict the QM/MM energies.^12−14^ This allows us to go beyond the standard QM/MM, for example, by fixing the artifacts at the boundary between QM and MM regions.^15^ However, apart from the need to tailor the ML potential to a given MM environment, these approaches often also go beyond the existing QM/MM architecture, by requiring additional information about the MM environment (beyond the point charges) or by introducing a more complex partitioning of the system. Therefore, these methods require not only retraining of the ML potentials but also changes in the QM/MM implementation.

A different approach was recently proposed by Zinovjev.^16^ There, instead of explicitly training on the QM/MM energy, the *in vacuo* energy of the QM part and the interaction between QM and MM (the embedding) are calculated by separate ML models. Below, we will refer to the model responsible for the embedding part of the total energy as “electrostatic ML embedding” or EMLE. A notable feature of EMLE is that it does not require QM/MM energies for training. Instead, it was shown that the response of the system to the presence of MM point charges can be described by learning the atomic properties of the QM system *in vacuo* and using analytic models of the electronic density and induction.^16^ This approach is based on previous work by Bereau et al.,^17^ where the interaction energy between two molecules was calculated based on their *in vacuo* properties, physics-based models, and just a handful of tunable parameters. The EMLE model could be seen as a limiting case of this approach, where one of the molecules is reduced to nonpolarizable point charges. The proposed ML electrostatic embedding scheme has two important advantages. First, it employs a separate *in vacuo* potential for the QM part. Therefore, if a pretrained (or generic) MLP for the QM subsystem of interest exists, it can readily be employed in the scheme, without further training. Second, the scheme only relies on the information that is usually provided to a QM engine in QM/MM software: the positions of the QM atoms and the positions and partial charges of the MM atoms. This allows to package the prediction code (*in vacuo* QM + embedding) as “just another QM engine” and directly use it with existing QM/MM software without significant modifications. These two advantages are explored in the present work.

The paper is structured as follows. First, we give a brief overview of the EMLE scheme. Second, we describe the ML/MM embedding implementation and show how it can be coupled to the sander program from the AmberTools23 package.^18^ Then, we apply the framework to calculate the free energy surface (FES) of alanine dipeptide in water using different options for the *in vacuo* and embedding models and compare the results to a reference hybrid DFT/MM FES. Finally, we discuss the obtained results and provide our perspective on future work.

## 2 Theory and Implementation

### 2.1 EMLE Scheme

Here, we give a brief description of the EMLE model; further details can be found in Zinovjev.^16^ The goal is to predict the total energy of a QM/MM (or MLP/MM) system treated with electrostatic embedding defined as
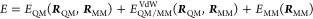
1where the three terms are the energy of the QM region polarized by including the interaction with the MM point charges, the van der Waals interaction between the two subsystems, and the MM energy. The last two terms are treated at the MM level and are therefore trivial to calculate with the corresponding force-field equations. In standard QM/MM calculations, the first term is calculated directly by determining the electronic energy in the presence of MM point charges, but this is not possible when using an MLP (in which electrons are not represented). EMLE aims to recover this first term without relying on an electronic description, by predicting the difference of the total energy of the QM (or MLP) subsystem *in vacuo* and in the presence of MM point charges:
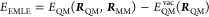
2resulting in the following expression for the total energy:
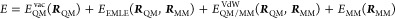
3

Physically, *E*_EMLE_ is the interaction energy between the MM point charges with the electrostatic potential of the polarized QM part plus the energetic cost of polarization. Since the electrostatic interaction is additive with respect to the electrostatic potential, it can be further decomposed into three components:
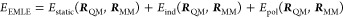
4where *E*_static_ is the electrostatic interaction energy between MM point charges and the unpolarized (gas phase) electronic density of the QM part, *E*_ind_ is the interaction between MM and the “induced electrostatic potential” (generated by the changes in the QM electronic density due to the presence of point charges), and *E*_pol_ is the polarization cost.

*E*_static_ is calculated by approximating the *in vacuo* electronic density of the QM part using minimal basis iterative stockholder (MBIS) partitioning.^19^ That is, the total density is described as the sum of atomic densities, consisting of a core charge (*q*^core^) and a valence Slater density characterized by the valence charge (*q*^val^) and the valence shell width (*s*):
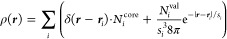
5where *N* = −*q* is the number of electrons in each shell. To take into account the long-range dependence of the atomic charges on the chemical structure, charge equilibration (QEq) was applied.^6,20^ QEq provides the total atomic charges (*q*_*i*_ = *q*_*i*_^core^ + *q*_*i*_^val^) by minimizing the following energy expression:
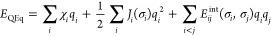
6where χ is the atomic electronegativity and *J* and *E*^int^ are the self-energy and interaction energy of unit Gaussian densities with width σ assumed to be proportional to the MBIS valence shell width *s* with the scaling factor *a*_QEq_:
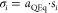
7

Then, *E*_static_ is calculated as the interaction energy between the MM charges *q*_*j*_ and the total electronic density:
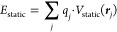
8

The induced potential and the polarization cost are obtained with the Thole model.^21^ Atomic polarizabilities are defined as being proportional to the MBIS atomic volumes:
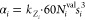
9where *k*_*Z*_*i*__ is the scaling factor different for each chemical element.

The induced atomic dipoles ***μ***_*i*_ are then obtained by solving the following system of linear equations:
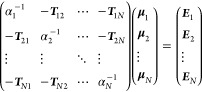
10where ***T***_*ij*_ are the dipolar interaction tensors with cubic exponential damping^21^ and ***E***_*i*_ are the screened electric fields caused by the MM point charges at the position of each QM atom. The screening is defined by considering the interaction between the MBIS atomic density and the external field:
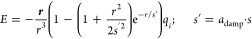
11

Here *a*_damp_ is the scaling factor for the valence shell widths that results in damping of the interaction. The value of 2 was found by a grid search (1.5–3 with 0.5 step) to be sufficient to avoid overpolarization, based on a comparison of (single point) induction energies in ML(EMLE)/MM and DFT/MM. Equation 11 is the only difference between the present work and EMLE as described in the original publication,^16^ where the interaction with the external field was calculated using atomic point dipoles. The damping is required to avoid overpolarization of the ML region.

Now, the total induction energy (including the interaction between induced dipoles and MM point charges as well as the polarization cost) can be calculated as half of the interaction between the MM point charges and the electrostatic potential formed by the induced dipoles:
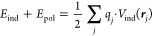
12where the factor 1/2 accounts for the polarization cost in the Thole model. Therefore, the sum of eq 8 and eq 12 gives the total embedding energy as defined in eq 4, with the polarization cost already being accounted for.

### 2.2 Implementation

The EMLE method was implemented in Python with the PyTorch^22^ machine learning framework used for the calculation of energies and gradients. A simple TCP (Transmission Control Protocol) socket server is used to handle requests for calculations from the client. This allows for a long-running process with access to the CPU/GPU resources throughout, thus avoiding the start-up cost of rerunning an executable, importing modules, etc., each time a calculation is required. In this case, the client is a “fake” *orca* executable that is used to intercept the external call by a QM/MM MD engine with an existing ORCA^23^ interface, then send a message to the EMLE server, and instruct it to perform a calculation. It is possible to use a single server to handle calculations from multiple clients, for example, in the case of limited resources, or to use a dedicated server for each client. The calculator used in the server supports the use of multiple backends for the calculation of *in vacuo* energies and gradients. Currently, we include support for the TorchANI,^24^ DeepMD,^25^ and rascal^26^ ML backends. We also support several QM backends (XTB, SQM, ORCA) and sander as the MM backend that can be combined with ML backends allowing for Δ-learning potential. For instance, in this work, sander with rascal Δ-learned correction was used. Support is also provided for user-defined external backends via an Atomic Simulation Environment^27^ calculator interface.

In this work, the open-source sander molecular dynamics engine from the AmberTools23 suite was used as the QM/MM MD engine.^28^ As described above, our code works as a drop-in replacement for the existing sander-ORCA QM/MM interface, i.e., it reads ORCA^23^ input files written by sander and writes outputs (energies and gradients) back to the ORCA format for sander to read.

## 3 Methodology

We apply the EMLE implementation described above to calculate the two-dimensional (2D) free energy surface (FES) of alanine dipeptide built as an alanine amino acid capped with an acetyl group on the N-terminus and *N*-methylamide on the C-terminus (ADP) in explicit water. ADP is treated with an MLP or QM method and water at the MM level (TIP3P). Various combinations of embedding schemes and *in vacuo* energy models are used. The results are compared to a reference FES at the DFT QM/MM (ωB97X/6-31G*/TIP3P) level^29^ to investigate the importance of different energy components for the accuracy of the resulting ML/MM potential.

### 3.1 System Setup and Relaxation

All the setup and relaxation steps were performed using the AmberTools23 package. The ADP molecule was initially described with the ff19SB force field^30^ and solvated in a box of TIP3P^31^ water molecules with at least 16 Å between the ADP and the box sides (with a total of 1948 water molecules). The system was prepared with tleap, and then the box was relaxed by running 100 ps of NPT MM MD with sander (Langevin thermostat, Berendsen barostat, 2 fs time step, with SHAKE restraints applied). The sander input is provided in the supporting GitHub repository.

### 3.2 Umbrella Sampling

The same Umbrella Sampling (US) protocol was used for all of the 2D FESs presented in this work. The backbone dihedral angles phi and psi were used as the reaction coordinates (RC). Across each angle, 36 windows were used, spaced by 10°, resulting in 1296 windows in total. The initial structures for each window were obtained at the MM level by first running 10 ps with a force constant of 10 kcal·mol^–1^·rad^–2^ and then 100 ps with the force constant of 100 kcal·mol^–1^·rad^–2^. The latter force constant was used for all of the subsequent US simulations, for both RCs. The sander input file with all the settings is provided in the supporting GitHub repository.

For all of the QM/MM and ML/MM simulations, 5 ps of sampling per window was obtained and integrated using WHAM.^32^ Such a short sampling time was chosen to limit the computational resources required for the reference 2D FES at the ωB97X/6-31G* level of theory. This functional was chosen for a fair comparison with the ANI-2x potential^33^ that used the same functional as the reference in training. All QM calculations were performed using ORCA 5.0.3.^23^ To estimate the statistical error of the resulting PMF, we performed 2D US at the MM level using 1 ns sampling per window, and the PMF was calculated both using the complete dataset (1 ns per window) and by splitting the windows into two hundred 5 ps chunks. The mean error of the bins from the resulting 200 PMFs with 5 ps sampling per window compared to the full PMF was 0.3 kcal·mol^–1^, which is below the error of the most precise of the tested ML/MM potentials (see below) and therefore justifies the usage of just 5 ps per window for the DFT reference and ML/MM models.

### 3.3 Tested Models

The architecture of the EMLE method and the proposed implementation allows us to combine different embedding and *in vacuo* models to estimate the importance of the energy components in the final combined potential. For the *in vacuo* ML potential, two models were used: (1) ANI-2x,^33^ which is a generic ML potential, and (2) an in-house trained potential specific for ADP conformational changes *in vacuo* (Δ-ML, see below). For embedding, three options were used: (1) the “MM” embedding model with the interaction between ML and MM being treated fully at MM level (using the fixed MM point charges from ff19SB for ADP, the ML region), (2) the “EMLE static” model where the induced term of eq 4 is neglected, and (3) the full generic electrostatic machine learning embedding model (or “EMLE total” model),^16^ which includes electrostatic and induction terms. All of the six combinations of the embedding and *in vacuo* models were tested to evaluate the impact of both the *in vacuo* and the interaction potentials on the quality of the free energy landscape of ADP in water.

### 3.4 Training of ADP-Specific Model

The *in vacuo* ADP-specific model (Δ-ML) was obtained using a delta-learning approach with the ff19SB MM energy as the low level of theory and ωB97X/6-31G* DFT as the reference. The Sparse Gaussian Approximation Potential with a quadratic SOAP kernel was used as the model architecture,^7^ and training was performed using the librascal package (https://github.com/lab-cosmo/librascal). The initial training set was obtained as follows. A single frame was taken from each window of the MM US calculation. For each frame, the ADP geometry was extracted and a short *in vacuo* DFT trajectory of 50 fs at 500 K was propagated, keeping the same RC restraints as in the corresponding US window. The elevated temperature is used to enhance the sampling of the conformational space. As a result, the bonds and angles are relaxed to the DFT potential. For the relaxed *in vacuo* ADP structures, single-point DFT calculations were performed, storing the total energy. The resulting 1296 structures with the corresponding DFT energies were used as the initial input to the iterative active learning procedure. At each iteration, the basis set of atomic environments for sparse Gaussian process regression (GPR)^34^ is selected with the informative vector machine (IVM) procedure.^35^ Then, the GAP potential is built by training on 80% of the structures in the training set with the prediction error being calculated over the remaining 20%. If the resulting error was below 1.5 kcal·mol^–1^, the potential was considered to be converged and the procedure stops. Otherwise, the trained potential is used to propagate short MD trajectories at 500 K (20–100 fs depending on the iteration). Single-point DFT calculations are then performed for the resulting structures and are added to the training set, and the procedure is repeated.

## 4 Results and Discussion

**Figure 1:**
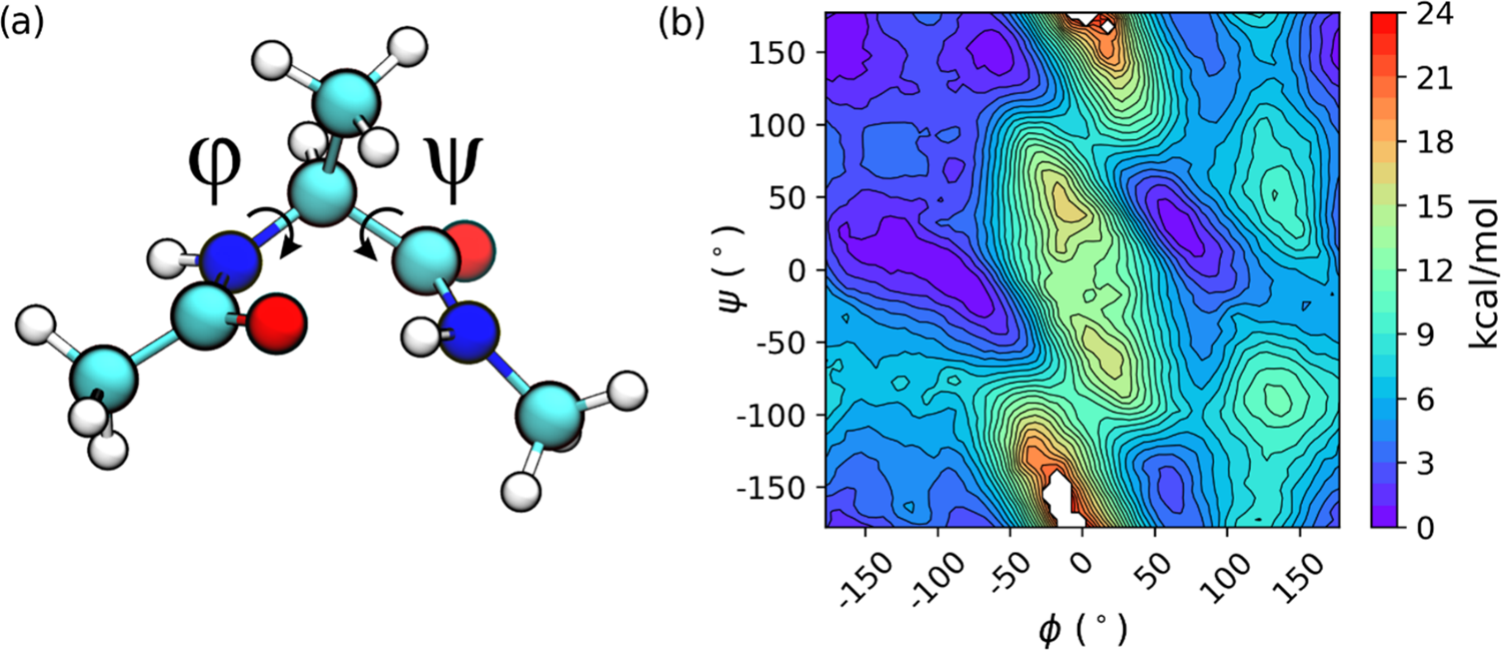
(a) Structure of ADP with the two backbone dihedrals used to define the FES. (b) ωB97X/MM FES.

The conformational landscape of ADP results forms a delicate balance between intra- and intermolecular interactions that depend on the environment.^36^*In vacuo*, the most populated ADP conformer is C7_eq_ (φ – 80°, ψ 75°)^37^ due to the formation of an intramolecular hydrogen bond interaction between the consecutive peptide groups. In an aqueous solution, the preferred conformer is P_II_ (φ – 70°, ψ 150°), with other states (C5 (φ – 150°, ψ 155°) and α_R_ (φ – 75°, ψ – 20°))^38^ also being populated due to the solute–solvent interactions favored by the larger accessibility of the polar groups.^36^Figure 1 shows the ADP model and the 2D DFT/MM free energy surface (FES) obtained in the aqueous solution using a standard electrostatic embedding scheme (eq 1). This FES qualitatively agrees with the experimental observations, showing that the preferred conformations in aqueous solution are α_R_ and P_II_. A more quantitative comparison would require longer simulations (the statistical error is estimated to be 0.3 kcal·mol^–1^; see Section 3.2) and integration over the surface to get the correct population of each conformer.

**Figure 2:**
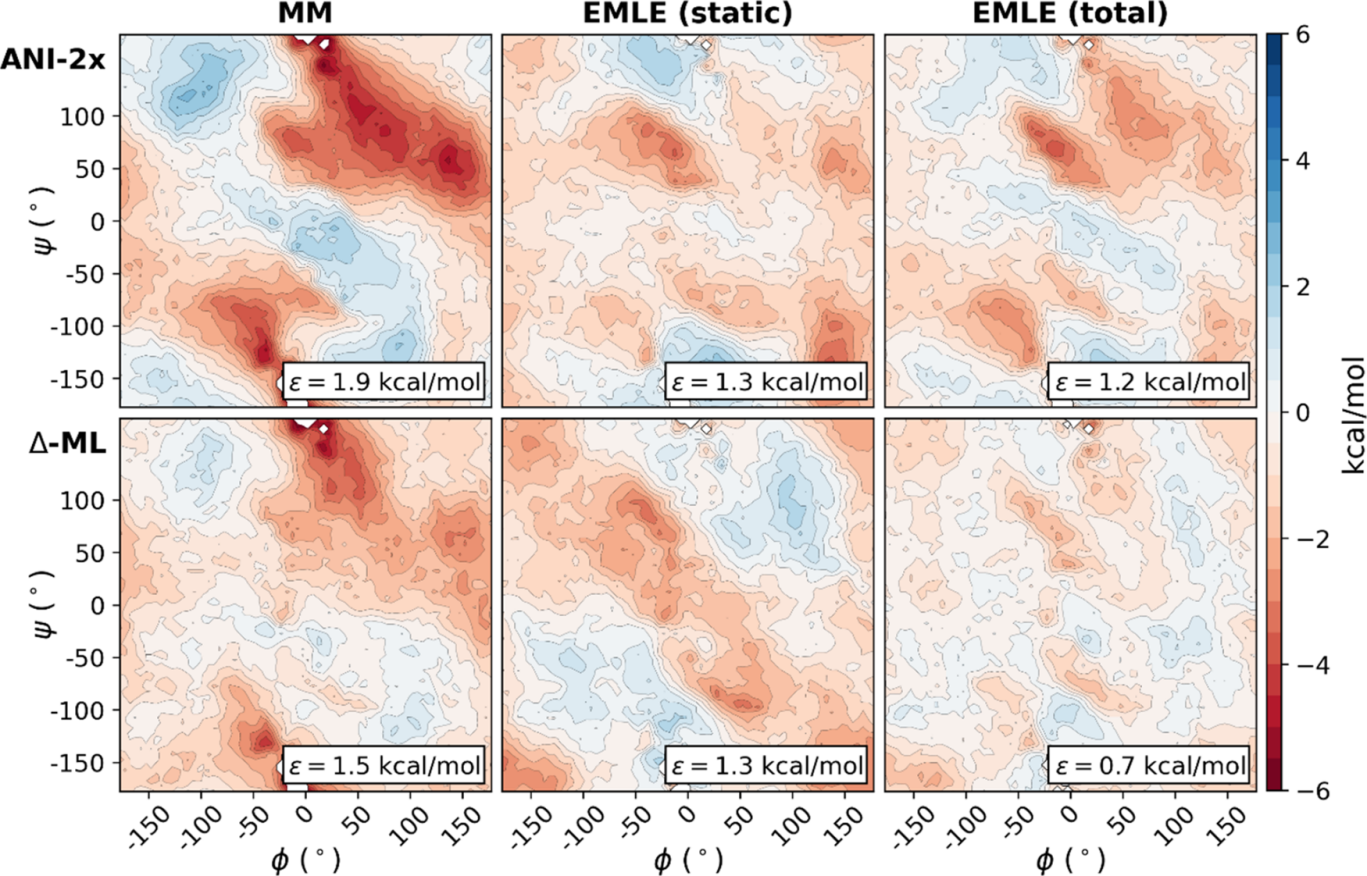
Differences between the FESs obtained at different ML/MM levels with respect to the reference DFT/MM (Figure 1). Above, FESs obtained using the ANI-2x *in vacuo* model and the three different embedding models (MM, EMLE static, and EMLE total). Below, FESs obtained using the *in vacuo* ADP-specific model (Δ-ML) and the three embedding models. The RMSE errors of each of the surfaces are provided.

The deviations of all of the four ML/MM FESs from the reference DFT surface are shown in Figure 2. The FESs are provided as Supporting Information (Figure S1). The error ε for each FES is calculated as the root mean square error (RMSE) over the FES bins. In all cases, only the bins with free energies below 20 kcal·mol^–1^ were considered to exclude bins that contain very few samples and therefore have a high statistical error.

The six ML/MM models each reproduce the main features of the DFT/MM surface (Figures 2 and S1) at a significantly reduced computational cost (see below), including capturing all of the preferred conformations of ADP discussed above. The RMSE values are, in all cases, smaller than 2 kcal·mol^–1^. The *in vacuo* potential has a significant impact on the total error. The ANI-2X generic ML potential results in RMSEs between 1.9 and 1.2 kcal·mol^–1^, depending on the embedding model. In contrast, using a system-specific Δ-ML potential reduces the RMSE almost by half (between 1.5 and 0.7 kcal·mol^–1^). This is not unexpected because the Δ-ML potential was specifically trained for the system studied. A comparison of the distribution of the potential energy errors of the two ML *in vacuo* potentials with respect to the ωB97X/6-31G* energies indicates that the Δ-ML potential reproduces the ωB97X/6-31G* energies better (Figure S2), which thus translates into a better description of the FES. The RMSE of Δ-ML + EMLE FES (0.7 kcal·mol^–1^) also gives the upper bound for the error of both the Δ-ML potential and EMLE, with most of the error coming from the embedding model (see Table S1).

The lack of significant improvement when the polarization component of EMLE is included with the ANI-2x potential is due to fortuitous error cancelation. The deviations of ANI-2x and EMLE (static) from the corresponding DFT/MM values correlate negatively (Table S1), thereby reducing the error of the resulting FES. The covariance analysis also reveals that the errors of MM embedding and the ANI-2x *in vacuo* potential are positively correlated. This results in an increased FES error for the ANI-2x + MM FES. A possible explanation is that both MM and ANI-2x potentials are primarily trained on ground-state structures and tend to have higher errors in transition states or other high-energy regions. This leads to an important observation: deficiencies in the *in vacuo* and embedding models accumulate if, for both, the same regions of conformational space are underexplored in the training set.

**Figure 3:**
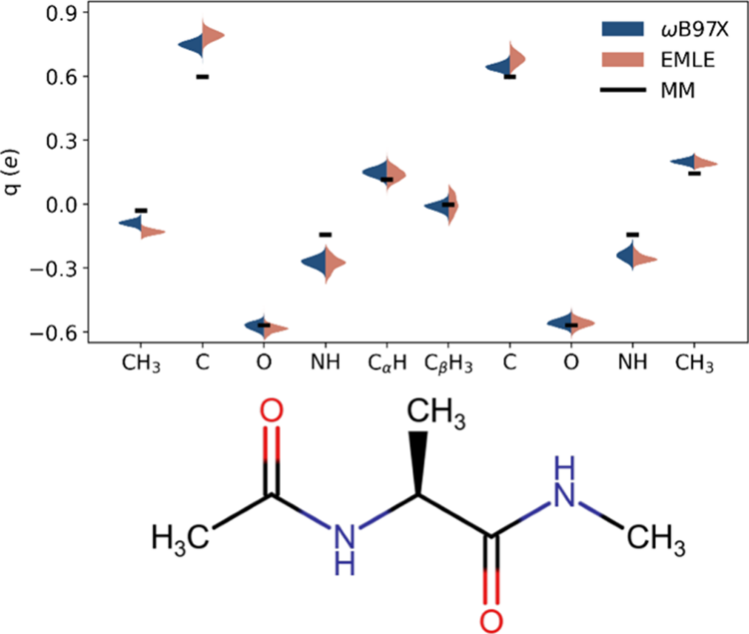
MBIS atomic charges calculated at the ωB97X/6-31G*/MM level and using EMLE, distributions accumulated over all windows in the FES. Hydrogen charges have been summed into heavy atoms. The MM charges (corresponding to the ff19SB force field) are shown for comparison.

Focusing on the embedding, for both the ANI-2x and the Δ-ML *in vacuo* potentials, we observe a reduction in the FES errors when moving from a simple MM embedding (fixed charges) to the EMLE embedding with only static electronic density (EMLE static) or to the full EMLE. The RMSE (ε) is reduced for the two *in vacuo* potentials when the MM embedding is changed to the EMLE static one: from 1.9 to 1.3 kcal·mol^–1^ in the ANI-2x calculations and from 1.5 to 1.3 kcal·mol^–1^ when using the specific Δ-ML potential for ADP (see Figure 2). These two embedding models consider only the interaction through partial charges, but while the MM embedding works with fixed point charges, in the EMLE static model, the atomic charges of ADP are spread into Slater functions and their magnitudes respond to geometrical changes in ADP. Figure 3 compares the distribution of MBIS charges calculated at the DFT level and with EMLE for ADP configurations sampled during the MD simulations, with hydrogen atom charges summed into their bonded heavy atoms (the ff19SB MM charges are also shown for comparison). The EMLE model is able to capture not only the average values of the DFT charges but also their fluctuations. An interesting observation is that the EMLE model differentiates between the two carbonyl carbon atoms, in agreement with DFT calculations, while these atoms are identical from the MM perspective. As demonstrated by the RMSE errors in Figure 2, the charge model implemented in EMLE provides a better description of the electrostatic interaction than the simpler fixed point charge model used in the MM force fields.

The improvement of EMLE-predicted partial charges over MM partial charges means that a unique set of nonpolarizable point charges is not enough to accurately reproduce the solute–solvent interaction energies for the whole set of structures that are being sampled during the conformational exploration. There are some ADP structures, far from the minimum energy regions (see regions of the FES with the largest errors in Figure 2), whose interaction energy with the solvent cannot be correctly represented with a single set of point charges. The conclusion is that even when only small changes are expected in the charge distribution of the solute (as for conformational change in the neutral ADP system), ML/MM embedding with fixed charges may fail to reproduce results from more accurate QM/MM simulations. The MM embedding is too rigid to describe solute–solvent interactions for conformations significantly different from those observed in or near the most populated minima. Instead, the flexible charges provided by the EMLE model improve the treatment of solute–solvent interactions significantly, even for ADP structures far from the most populated conformations (Figure 2). The use of a flexible embedding model thus appears to be a requisite for the correct treatment of solute–solvent interactions when a variety of configurations, with different charge distributions, are considered. This is a key observation for the development of ML/MM strategies for simulating chemical processes, in particular when the charge distribution of the solute or reacting species can show significant variations, e.g., in a chemical reaction.

**Figure 4:**
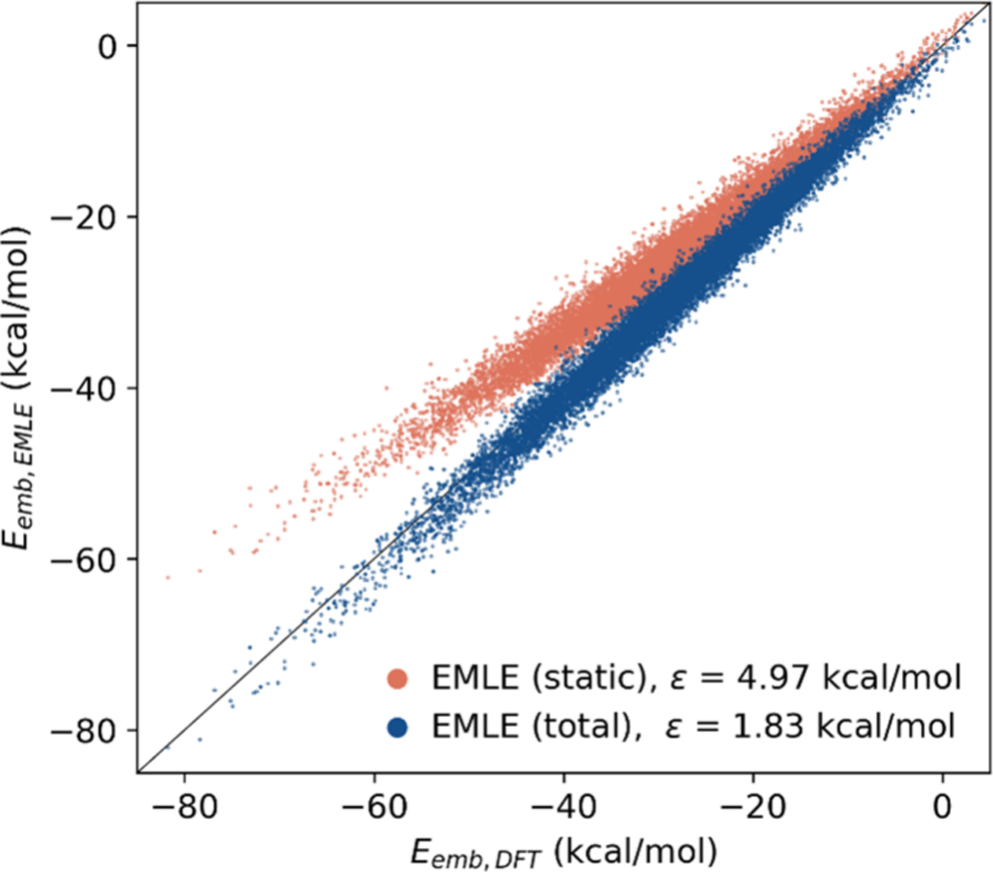
Embedding energies obtained with both static and total EMLE models versus the reference DFT values. The errors are calculated as RMSE with respect to the DFT values.

In spite of the previous analysis, it must be stressed that MM predicts the full embedding energies better than the static EMLE model but worse than the full EMLE model with charges and polarizabilities (RMSEs of 2.47, 4.97, and 1.83 kcal·mol^–1^, respectively, see Table S1). This trend is not surprising, considering that the MM charges are developed to implicitly include polarization effects.^39^ Inclusion of ADP polarizability in the embedding model further reduces the RMSE with respect to the DFT/MM result both in the total and in the embedding energies (see Table S1 and Figure 2). The effect of the induction energy is more perceptible when using the Δ-ML potential for ADP, which provides a better description of the *in vacuo* potential. A larger error of the underlying ML potential can mask errors in the embedding model by means of a partial error cancelation (Table S1). The inclusion of the induction term is important, in principle, because it can vary greatly between different configurations (even for ADP, where the changes in the interaction between the solute and the solvent are relatively small). In contrast to the charges used to calculate the static part of the embedding energy, which depend solely on the solute’s geometry, the induction component depends also on changes in the environment. When comparing the static and total EMLE embedding energies obtained for the ADP configurations to the DFT QM/MM embedding energies (Figure 4), the EMLE model correctly describes the total interaction energy and the contribution of polarization. A near-perfect correlation between the EMLE and QM/MM embedding energies is also maintained when the induction energy term becomes larger, i.e., for those configurations with a larger solute–solvent interaction. It must be stressed that an embedding model describing polarization correctly is essential to discriminate the interaction of a solute (or small molecule) with different environments, for instance, for the evaluation of free energies of binding or transfer. It can also be particularly important in metalloenzymes, where the substrate–metal interaction and polarization can be very intense. An important example is ATPases, enzymes hydrolyzing ATP, that bind Mg^2+^ and ATP phosphate groups in close proximity, two highly charged species. The correct description of the interaction between these two ions requires the inclusion of polarization effects.^40^ Although more research is clearly needed, the EMLE embedding model is a promising tool for the correct description of such difficult cases.

### 4.1 Performance Analysis

Using a single thread of an Intel Skylake CPU clocked at 1 GHz, *emle-engine* currently runs the ADP system (ADP + solvent box, 22 + 5844 atoms) at ∼145 ps/day, with ADP modeled using ANI-2x (and sander for propagation). In comparison, the performance of reference DFT/MM calculations is 0.44 ps/day, approximately 300-fold slower than ML/MM MD with *emle-engine*. Performance-wise, this makes our current implementation close to that of QM/MM with efficient semiempirical methods. For example, GFN2-xTB^41^ via the external ORCA interface (in combination with sander) gives a performance of ∼290 ps/day on the same hardware. For larger MLP subsystems, we expect *emle-engine* to take better advantage of parallelization and become more efficient than semiempirical QM methods.

## 5 Conclusions

We have presented a practical implementation of an ML-based electrostatic embedding model to carry out molecular dynamics simulations with hybrid ML/MM schemes. The developed *emle-engine* package is based on the EMLE embedding scheme, which predicts the ML–MM interaction energy using physics-based models.^16^ The static component of the electrostatic interaction energy is calculated by approximating the *in vacuo* electron density of the embedded subsystem using MBIS partitioning. The induction component is obtained from the Thole model. This functional form incorporates the essential physics of the QM/MM interaction energy with electrostatic embedding and therefore makes the model generic, despite relying on only a handful of free parameters.

*emle-engine* was tested on the calculation of the conformational free energy surface of ADP in an aqueous solution. We have obtained the surfaces corresponding to two different *in vacuo* ML potentials and three embedding options: MM embedding using fixed MM point charges, the static component of the EMLE model, which only includes the electrostatic component, and the total EMLE model, which includes electrostatic and polarization terms. All simulations resulted in stable trajectories for all umbrella sampling windows. Comparison of the results obtained for the six models to the reference ωB97X/MM surface shows that the EMLE embedding systematically reduces the average errors compared to MM embedding, with the latter not able to fully capture changes in the solute–solvent interaction energies associated with the conformational change. EMLE significantly improves the treatment of solute–solvent interactions due to the dependence of the electronic density on the solute’s geometry and the inclusion of the induction and polarization energy, which also depends on the configuration of the environment. Comparison between EMLE and DFT embedding energies shows that our model also correctly captures polarization effects, which can play an important role to discriminate among different environments. EMLE embedding is thus a promising model for ensuring an adequate treatment of the interaction between ML and MM subsystems, including for cases where such interactions are crucial to include, such as for simulating catalysis of chemical reactions, where the charge distribution of the reacting species changes significantly. The proposed framework is flexible enough to capture the changes in the interaction energy with the environment in such cases. The EMLE model can be easily fitted to work in any existing QM/MM framework: the QM energy can be substituted by the sum of the *in vacuo* MLP energy (depending only on the coordinates of the ML subsystem) and the interaction energy provided by EMLE, which is a function of both the ML and MM coordinates.

We conclude that the proposed *emle-engine* package provides stable ML/MM MD trajectories, can be used with existing QM/MM codes, and is compatible with arbitrary *in vacuo* ML potentials. We also show that the EMLE model is sufficiently flexible to capture changes in the electronic interaction between the embedded subsystem and the surroundings, paving the way for the use of MLP/MM for accurate simulations of chemical processes in condensed phases.

## Data Availability

Example input files for the performed simulations as well as the data and scripts used to generate all of the figures in this publication are provided in the supporting GitHub repository (https://github.com/chemle/emle-engine-paper).

## Supporting Information Available

ADP Free energy surfaces for the six ML/MM potentials used (Figure S1); distribution of the energy prediction errors with the two *in vacuo* ML potentials used (Figure S2); covariance and correlation between errors of *in vacuo* potentials and embedding models (Table S1) (PDF)
