# Supplementary material for: emle-engine: A Flexible Electrostatic Machine Learning Embedding Package for Multiscale Molecular Dynamics Simulations
Source: J Chem Theory Comput. 2024 May 28;20(11):4514–22. doi: 10.1021/acs.jctc.4c00248 (PMC11171281; doi:10.1021/acs.jctc.4c00248)
Supplement: Supplementary file 1 — ct4c00248_si_001.pdf [file ct4c00248_si_001.pdf]

Supplementary Information

for

emle-engine: a flexible electrostatic machine learning embedding package for multiscale molecular dynamics simulations

Kirill Zinovjev[*,1], Lester Hedges,[2,3], Rubén Montagud Andreu[1], Christopher Woods[3], Iñaki Tuñón[1], Marc W. van der Kamp[*,2]*

[1] Departamento de Química Física, Universidad de Valencia, 46100, Burjassot (Spain)

[2] School of Biochemistry, University of Bristol, Biomedical Sciences Building, University Walk, Bristol BS8 1TD, UK

[3] Research Software Engineering, Advanced Computing Research Centre, 31 Great George Street, Bristol BS1 5QD, UK

[*]to whom correspondence should be addressed: kirill.zinovjev@uv.es; marc.vanderkamp@bristol.ac.uk

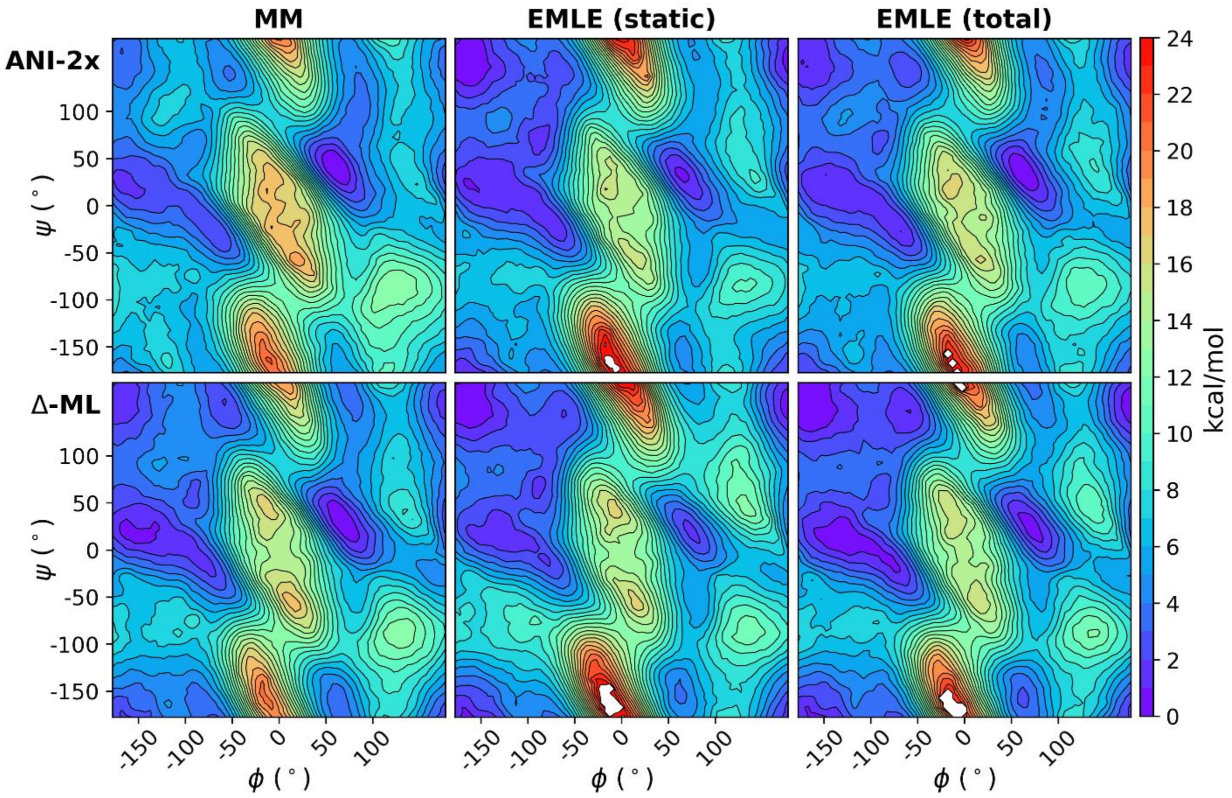

Figure S1. ADP Free energy surfaces for the six ML/MM potentials used. Above, FESs obtained using the ANI-2x in vacuo model and the three different embedding models (MM, EMLE static and EMLE total). Below, FESs obtained using the *in vacuo* ADP-specific model (Δ-ML) and the three embedding models.

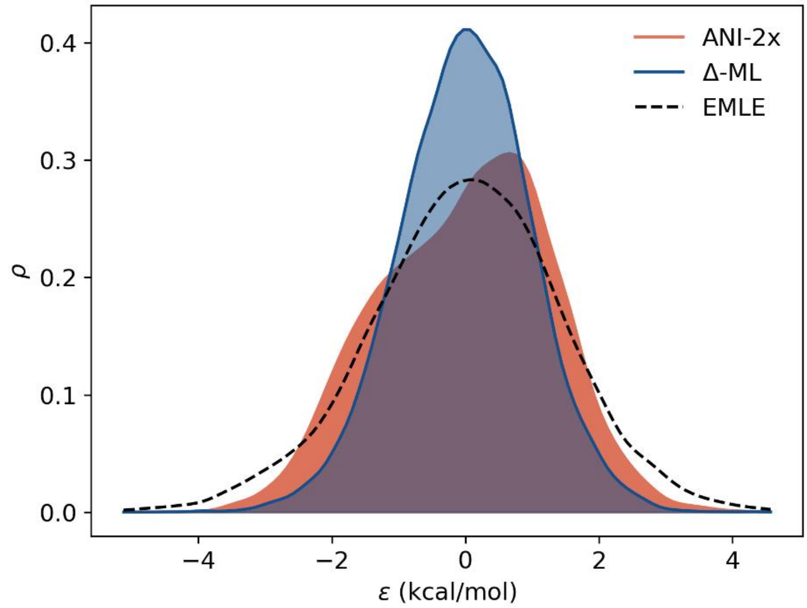

Figure S2. Distribution of potential energy errors obtained with the two *in vacuo* ML potentials (ANI-2x and the specific Δ-ML potential) w.r.t the ωB97X/6-31G* energies and of EMLE w.r.t the ωB97X/6-31G*//MM energies.

Table S1. Covariance ($kcal^2 \cdot mol^{-2}$) / correlation between errors (w.r.t. reference DFT calculations) of *in vacuo* potentials and embedding models used. The value in parentheses is the RMSE of the models w.r.t. the corresponding (*in vacuo* or embedding) reference values ($kcal \cdot mol^{-1}$).

	**MM (2.47)**	**EMLE static (4.97)**	**EMLE total (1.83)**
**ANI-2x (1.27)**	0.65 / 0.26	-0.38 / -0.11	0.07 / 0.04
**Δ-ML (0.98)**	-0.09 / -0.05	-0.07 / -0.03	-0.11 / -0.08
